# Metagenomic analysis of fecal microbiomes reveals genetic potential for diverse hydrogen management strategies in marsupials

**DOI:** 10.1128/msystems.01608-25

**Published:** 2025-12-23

**Authors:** Kate L. Bowerman, Yang Lu, Harley McRae, James G. Volmer, Julian Zaugg, Phillip B. Pope, Philip Hugenholtz, Chris Greening, Mark Morrison, Rochelle M. Soo, Paul N. Evans

**Affiliations:** 1 Australian Centre for Ecogenomics, School of Chemistry and Molecular Biosciences, The University of Queensland 1974, Brisbane, Australia; 2 Water Innovation and Smart Environment Laboratory, School of Civil and Environmental Engineering, Faculty of Engineering, Queensland University of Technology 1969, Brisbane, Australia; 3 Frazer Institute, Faculty of Health, Medicine and Behavioural Sciences, The University of Queensland 1974, Brisbane, Australia; 4 Centre for Microbiome Research, School of Biomedical Sciences, Queensland University of Technology 1969, Brisbane, Australia; 5 Faculty of Chemistry, Biotechnology and Food Science, Norwegian University of Life Sciences, Ås, Norway; 6 Department of Microbiology, Biomedicine Discovery Institute, Monash University 161661, Clayton, Australia; University of Connecticut, Storrs, Connecticut, USA

**Keywords:** gut microbiome, marsupials, methanogenesis, ruminants, hydrogen sinks

## Abstract

Methane is an end product of plant biomass digestion by gut microbiota, though the amount produced and/or released varies between hosts. On a per-unit-of-feed basis, macropodid marsupials (e.g., kangaroos) have been reported to emit less methane than ruminant livestock, despite a similar diet, although measurements exist for only a subset of macropodid species. Competition for hydrogen within the gut microbiome, particularly through alternative hydrogen sinks to methanogenesis, influences methane production; therefore, characterizing hydrogen management strategies within a host system can provide insights into methane emission profiles. In this study, we analyzed 33 fecal microbiomes of 14 marsupial species (predominantly captive animals) to provide the first systematic characterization of methanogen types and hydrogen-cycling genetic capacity across marsupial gut microbiomes. We recovered 1,394 metagenome-assembled genomes and identified host-associated bacterial signatures that varied significantly between marsupial species. Comparative analysis with fecal microbiomes from high- and low-methane-emitting mammals revealed that marsupials display heterogeneous hydrogen management strategies: some harbor elevated methanogenesis genes (mcrA, methanogen-specific hydrogenases), while others show enrichment of bacterial hydrogen-uptake hydrogenases and alternative electron acceptor pathways (nitrate/nitrite reduction, sulfite reduction). This predicted functional variation occurs both between and within marsupial families and gut types, suggesting that hydrogen management capacity may differ within taxonomic and anatomical classifications. These results demonstrate that marsupial gut microbiomes cannot be treated as a functionally homogenous group regarding methane emissions and highlight the need for species-specific measurements to accurately assess their methanogenic potential and inform ecological models of greenhouse gas production.

**IMPORTANCE:**

Herbivorous marsupials such as kangaroos and wallabies have been reported to produce significantly lower methane emissions than ruminant livestock despite eating a similar diet, yet the microbial mechanisms underlying this difference remain poorly understood. Here, we conduct a comparative study of fecal microbiomes of 14 marsupial species to provide the first investigation of hydrogen-cycling genetic capacity across these animals. Through comparative analysis with fecal microbiomes of high- and low-methane-producing animals, we identify enrichment of bacterial genes for alternative hydrogen uptake and disposal pathways in some marsupials, supporting competition for hydrogen playing a role in the level of methane production. These data also indicate variation in hydrogen management between marsupials, including within species, suggesting methane emission capacity may vary at the level of the individual.

## INTRODUCTION

Hydrogen management is a key component shaping gut microbiome community structures (1). Gut bacteria produce hydrogen during fermentation of plant biomass, requiring the activity of hydrogen-consuming organisms to maintain the low hydrogen partial pressure necessary for fermentation to be energetically favorable (2). While methanogenic archaea represent one major hydrogen sink, coupled to their production of methane, alternative pathways such as reductive acetogenesis, sulfate reduction, and nitrate ammonification compete with methanogenesis for available hydrogen (3, 4). The balance between these pathways and methane production varies substantially across different species, influenced by differences in gut physiology and diet; however, many hosts remain uncharacterized (5, 6).

Marsupials display diverse digestive physiology, including foregut-fermenting species, such as kangaroos and wallabies (macropodids), and hindgut fermenters including wombats and koalas (7). Quantitative measurements from four macropodid species revealed these animals emit 4- to 10-fold lower methane compared to foregut-fermenting ruminant livestock per unit of equivalent feed (6, 8–11). This apparent low-methane phenotype has been attributed to the shorter gastrointestinal tract and lower retention time of digesta relative to ruminants, resulting in decreased hydrogen levels that limit methane production (9, 12). However, increased abundance of fermentative bacteria that produce succinate and/or propionate as an electron sink without producing hydrogen may also play a role, for example, the large populations of *Succinivibrionaceae* associated with reduced methane emissions in the Tammar wallaby (13). While methanogenic archaea have been detected in both the macropodid foregut and in fecal samples from hindgut-fermenting marsupials at low abundance (14–17), their metabolic capabilities and substrate preferences are unclear, as is the extent to which alternative hydrogen-consuming bacteria compete with methanogens across different marsupial lineages.

Recent genome-resolved metagenomic studies of ruminants have revealed that microbial community differences in hydrogen management capacity provide a genetic basis for variation in methane production levels between high- and low-methane animals (3, 18). Low-methane cattle show enrichment of bacterial genes encoding membrane-bound hydrogen-uptake hydrogenases for hydrogenotrophic respiration, reductant disposal pathways including propionate and butyrate production, and terminal reductases for nitrate and sulfate reduction, while high-methane cattle show greater abundance of fermentative hydrogen-producing hydrogenases and methanogenesis genes (18). Similar shifts toward succinate, lactate, and propionate production have been associated with low-methane-emitting cattle and sheep (19–23). Conversely, reduced methane production can also reflect the increased activity of hydrogen-consuming bacteria that reduce the electron pool available for methanogens. Indeed, recent studies have suggested ruminants harbor novel lineages of acetogenic bacteria and various hydrogenotrophic respiratory microbes that use electron acceptors such as fumarate, nitrate, and sulfate (3, 23–26). These findings suggest that characterizing the genetic complement of hydrogen-cycling genes can reveal the mechanistic basis for alternative hydrogen management strategies, even when active metabolic rates cannot be directly measured.

Here, we use genome-resolved metagenomic analyses of 33 fecal microbiomes of 14 marsupial species, from predominantly captive animals, to investigate the key pathways and mediators of hydrogen cycling and methane formation in these animals. We employ comparative analysis against fecal microbiomes of high- and low-methane-emitting mammals of varied gut types to identify differentially enriched hydrogen-metabolizing genes and pathways. Through recovery of 1,394 metagenome-assembled genomes (MAGs) and functional annotation of methanogens and hydrogen-cycling genes, including hydrogenases, terminal reductases, and enzymes for alternative fermentation products, we provide the first characterization of genetic hydrogen management capacity across marsupial fecal microbiomes and extend the framework for understanding microbial contributions to methane production diversity in herbivorous mammals.

## MATERIALS AND METHODS

### Sample collection

Fecal samples were collected from 14 species of marsupial from predominantly captive environments in Queensland, Australia (Table 1; Table S1). Species included were the fat-tailed dunnart (*Sminthopsis crassicaudata*, *n* = 3), Lumholtz’s tree kangaroo (*Dendrolagus lumholtzi*, *n* = 2), eastern gray kangaroo (*Macropus giganteus*, *n* = 1), red-legged pademelon (*Thylogale stigmatica*, *n* = 1), northern brown bandicoot (*Isoodon macrourus*, *n* = 2), yellow glider (*Petaurus australis*, *n* = 2), mahogany glider (*Petaurus gracilis*, *n* = 2), squirrel glider (*Petaurus norfolcensis*, *n* = 5), koala (*Phascolarctos cinereus*, *n* = 4), rufous bettong (*Aepyprymnus rufescens*, *n* = 2), long-nosed potaroo (*Potorous tridactylus*, *n* = 2), greater glider (*Petauroides volans*, *n* = 2), southern hairy-nosed wombat (*Lasiorhinus latifrons*, *n* = 4), and common wombat (*Vombatus ursinus*, *n* = 1). Fecal samples were stored at −80°C as soon as possible following collection. Public data sets incorporated into this study are available under NCBI BioProjects PRJEB50625 (27), PRJNA752224 (28), PRJNA987743 (29), PRJNA1102860 (30), SRP012966 (31), and PRJNA340521 (32). Only fecal samples were included (Table S2).

**TABLE 1:** Samples used in this study

Sample	Species	Common name	Location
DUN1	*Sminthopsis crassicaudata*	Fat-tailed dunnart	Currumbin Wildlife Sanctuary
DUN2	*Sminthopsis crassicaudata*	Fat-tailed dunnart	Currumbin Wildlife Sanctuary
DUN3	*Sminthopsis crassicaudata*	Fat-tailed dunnart	Currumbin Wildlife Sanctuary
LTK1	*Dendrolagus lumholtzi*	Lumholtz’s tree kangaroo	Currumbin Wildlife Sanctuary
LTK2	*Dendrolagus lumholtzi*	Lumholtz’s tree kangaroo	Wongabel State Forest (wild)
KEG1	*Macropus giganteus*	Eastern-gray kangaroo	Lone Pine Koala Sanctuary
RLP1	*Thylogale stigmatica*	Red-legged pademelon	Lone Pine Koala Sanctuary
BAN1	*Isoodon macrourus*	Northern brown bandicoot	Hidden Vale Research Station
BAN2	*Isoodon macrourus*	Northern brown bandicoot	Hidden Vale Research Station
YEG1	*Petaurus australis*	Yellow glider	Currumbin Wildlife Sanctuary
YEG2	*Petaurus australis*	Yellow glider	Currumbin Wildlife Sanctuary
MHG1	*Petaurus gracilis*	Mahogany glider	Wildlife Habitat
MHG2	*Petaurus gracilis*	Mahogany glider	David Fleay Wildlife Park
SQG2	*Petaurus norfolcensis*	Squirrel glider	Currumbin Wildlife Sanctuary
SQG3	*Petaurus norfolcensis*	Squirrel glider	Currumbin Wildlife Sanctuary
SQG4	*Petaurus norfolcensis*	Squirrel glider	Hidden Vale Research Station
SQG5	*Petaurus norfolcensis*	Squirrel glider	Hidden Vale Research Station
SQG6	*Petaurus norfolcensis*	Squirrel glider	Hidden Vale Research Station
KOA1	*Phascolarctos cinereus*	Koala	Currumbin Wildlife Sanctuary
KOA2	*Phascolarctos cinereus*	Koala	Lone Pine Koala Sanctuary
KOA3	*Phascolarctos cinereus*	Koala	Wildlife Habitat
KOA4	*Phascolarctos cinereus*	Koala	Cairns Tropical Zoo
BET1	*Aepyprymnus rufescens*	Rufous bettong	Hidden Vale Research Station
BET2	*Aepyprymnus rufescens*	Rufous bettong	Hidden Vale Research Station
POT1	*Potorous tridactylus*	Long-nosed potaroo	Hidden Vale Research Station
POT2	*Potorous tridactylus*	Long-nosed potaroo	Hidden Vale Research Station
GRG1	*Petauroides volans*	Greater glider	Currumbin Wildlife Sanctuary
GRG2	*Petauroides volans*	Greater glider	Currumbin Wildlife Sanctuary
SHN1	*Lasiorhinus latifrons*	Southern hairy-nosed wombat	Currumbin Wildlife Sanctuary
SHN2	*Lasiorhinus latifrons*	Southern hairy-nosed wombat	Currumbin Wildlife Sanctuary
SHN3	*Lasiorhinus latifrons*	Southern hairy-nosed wombat	Cairns Tropical Zoo
SHN4	*Lasiorhinus latifrons*	Southern hairy-nosed wombat	Lone Pine Koala Sanctuary
WOM1	*Vombatus ursinus*	Common wombat	Lone Pine Koala Sanctuary

### Sample preparation and metagenomic sequencing

Total genomic DNA was extracted from approximately 60 mg–200 mg of fecal material. Samples were homogenized at 2,000 rpm for 5 min using the MoBio PowerLyzer24 in a MoBio bead tube containing 0.1 mm diameter zirconia/silica beads and 750 µL of TLA buffer (Promega, WI, USA). The supernatant was recovered via centrifugation at 10,000 *g* for 30 s. DNA was extracted from 150 µL of supernatant using the Maxwell 16 robotic system and the corresponding Tissue DNA kit (Promega, WI, USA) following the manufacturer’s instructions.

Illumina sequencing libraries were prepared using the Nextera DNA Library Preparation kit (Illumina, CA, USA). Samples were sequenced on the NovaSeq 6000 platform, with some samples supplemented with data generated using the HiSeq 2000 platform, producing ~7 Gb of 150 bp paired-end reads per sample.

Oxford Nanopore sequencing was performed on a PromethION instrument using an R10.4.1 flow cell following sample preparation with the Ligation Sequencing Kit SQK-NBD114-24 (Oxford Nanopore, UK). Samples were multiplexed using native barcoding and run using adaptive sampling, generating ~5 Gb per sample with an average read length of 800 bp. Five archaeal MAGs were used as a positive filter for adaptive sampling using MinKNOW v.23.07.5. Enrichment of archaeal populations was estimated by comparison of their relative abundance between Illumina and Nanopore sequencing runs (Table S3). Guppy v.7.0.9 was used for initial base-calling in high-accuracy mode. Subsequent recalling to super-high accuracy was undertaken using Dorado v.0.5.2 (https://github.com/nanoporetech/dorado) following pod5 conversion with pod5 v.0.3.10 (https://github.com/nanoporetech/pod5-file-format).

### Data assembly

Illumina data were quality trimmed and cleaned of adapters using Trimmomatic v.0.39 (33). Read counts pre- and post-trimming for all data were generated using SeqKit v.2.4.0 (34). The trimmed reads were then assembled with MEGAHIT v.1.2.9 (35). Reads were mapped to assembled contigs using BamM (https://github.com/Ecogenomics/BamM) and binned using MetaBAT2 v.2.15 (36).

Sequence barcodes were removed from Nanopore sequence data using Porechop v.0.2.4 (https://github.com/rrwick/Porechop), and reads with mid-strand barcodes were also removed. Hybrid assembly of Nanopore and Illumina data from the same samples was undertaken using Aviary v.0.8.3 (https://github.com/rhysnewell/aviary) with default parameters. Binning was performed by Aviary v.0.8.3 employing Rosella v.0.5.3 (https://github.com/rhysnewell/rosella), MetaBAT1 and MetaBAT2 v.2.15 (36), and CONCOCT v.1.1.0 (37) based on read mapping of all five Nanopore-targeted samples to each assembly. DAS Tool v.1.1.6 (38) was used to determine a non-redundant MAG set.

All MAGs were quality assessed using CheckM v.1.2.2 (39) and CheckM2 v.1.0.2 (40), and classified using GTDB-Tk v.2.3.0 (41) against GTDB releases 08-RS214 and 09-RS220 (42, 43). Nanopore- and Illumina-based MAGs exceeding 50% completeness with ≤5% contamination according to either CheckM or CheckM2 were combined and dereplicated at 99% average nucleotide identity (80% alignment fraction) using CoverM v.0.7.0 (44) applying the “Parks2020_reduced” quality formula.

### Genome database

Public genomes to be included in the final genome database were selected from those within GTDB release 08-RS214 (42, 43) based on relative abundance and coverage values generated using CoverM v.0.7.0 (44). Genomes were included that achieved a relative abundance of >0.05% and >0.1× coverage in at least one sample. Selected genomes were combined with the dereplicated sample MAGs (≥50% complete with ≤5% contamination), and the combined set was dereplicated at 95% average nucleotide identity (60% alignment fraction) using CoverM v.0.7.0, applying the “Parks2020_reduced” quality formula. Community profiling was undertaken using relative abundance values generated from mapping short-read data to this database using CoverM v.0.7.0 “genome.” BWA (45), implemented within CoverM, was used for Illumina read mapping with the parameter “-k 31,” while Minimap2 (46) option “-p minimap2-hifi” was used for Nanopore data (used for host DNA estimation and evaluation of adaptive sequencing only). Read alignments were filtered for those achieving ≥95% identity across ≥90% of the read length. Genomes were functionally annotated using DRAM v.1.5.0 (47) with the KOfam (48), Pfam (49), and dbCAN (50) databases, accessed 21 February 2024. Contigs not recruited into MAGs were compiled for assessment of the taxonomic composition of unmapped reads using MMseqs2 v.15-6f452 (51) “easy-taxonomy” against the NCBI NR protein database (52) for taxonomic inference.

### Host tree

The marsupial host phylogenetic tree was inferred using three nuclear (*IRBP, BRCA1, vWF*) and two mitochondrial genes (*CYTB, ND2*), which were selected based on sequence availability (Table S4). Sequences were aligned using MAFFT v.7.490 (53) and trimmed of gaps present in >10% of sequences using trimAl v.1.4.1 (54). Maximum-likelihood trees were inferred using IQ-TREE v.2.2.2.3 (55), using ModelFinder for model selection with gene and codon partitioning. Bootstrap support was generated from 100 replicates.

### Host DNA estimation

An estimate of host DNA contamination was created using available marsupial genomes in combination with a set of exon capture sequences (56) (Table S5). Due to the absence of full-length genomes for all hosts, this analysis is considered to provide only a minimum estimate of host DNA contamination. Read mapping was undertaken using BWA (45), as above. Estimated host DNA was calculated based on read numbers aligning with ≥95% identity across ≥90% of the read length, determined using CoverM v.0.7.0.

### Marker gene-based community profiling

SingleM v.0.16.0 (57) was used for marker gene-based community profiling using the GTDB release 08-RS214 metapackage, with the “condense” function used to summarize data across markers, and SingleM microbial_fraction used to estimate the bacterial and archaeal community fraction (58).

### Protein database creation and functional annotation

The gene database was created from proteins within assembled contigs from marsupial and ruminant samples annotated using Prodigal v.2.6.3 (59). Proteins were clustered at 90% identity across 80% of protein length using MMseqs2 v.14-7e284 (51). Representative protein sequences were functionally annotated using DRAM v.1.5.0 (47) with the KOfam (48), Pfam (49), and dbCAN (50) databases accessed 21 February 2024.

Protein abundance within samples was calculated based on read alignment using DIAMOND v.2.1.0 (60) to the protein database, filtering for sample reads ≥140 bp with Seqkit v.2.4.0 (34) (excepting public data set PRJNA987743, where read length was 120 bp). Alignments were filtered using settings evalue 0.00001, min-score 40, query-cover 80, id 70, max-hsps 1, and max-target-seqs 1. Alignment of reads to the single-copy marker genes included within the SingleM GTDB release 08-RS214 metapackage (57) was undertaken using the same settings. Reads per kilobase per million mapped reads (RPKM) values were calculated for both the protein database and SingleM marker genes, with the mean RPKM value across all SingleM markers used to normalize protein database values on a per-sample basis. Normalized RPKM values per protein were summed across equivalent functional annotations (e.g., KEGG orthologs or hydrogenase groups) and log_2_-transformed for PCA and differential abundance analysis.

### Hydrogenase classification

Pfam annotations from DRAM were used to collate a set of hydrogenase proteins present in the protein database or in MAGs based on hydrogenases listed in Greening et al. (3) (Table S6). Collated protein sequences from the protein database were clustered at 50% identity, and representatives were submitted to HydDB for classification (61). The taxonomic origin of hydrogenases (and KOs of interest) was inferred using MMseqs2 v.15-6f452 (51) “easy-taxonomy” on the containing contigs (database: GTDB 08-RS214).

### Methanogenesis pathway analysis

Archaeal genomes were annotated using DRAM v.1.5.0 (47). KEGG module presence was determined using EnrichM v.0.6.1 (https://github.com/geronimp/enrichM) with custom modules describing sub-components of methanogenesis pathways (Table S7). Candidate missing enzymes were identified using BLAST (62) with characterized reference sequences from UniProt (63).

### Statistical analyses and figure generation

PCA was conducted using vegan v.2.6-4 (R v.4.3.2) (64) using Hellinger-transformed data following filtering for taxa present in ≥2 samples. Contributing variables were filtered and plotted using factoextra v.1.0.7 (R v.4.3.2) (65). All other R-based analyses were conducted using R v.4.4.0. Redundancy analysis was conducted using the “rda” function within vegan v.2.6-6.1, with significance determined using “anova.cca” (64). For PCA and redundancy analysis incorporating public data sets, a maximum of 10 samples per host species were included.

Differential abundance analysis was undertaken using sPLS-DA implemented within mixOmics v.6.28.0 (66) based on centered-log ratio transformed relative abundance values (community profiles) or log_2_-transformed RPKM values (gene profiles). Community profile data were filtered for taxa present in ≥2 samples. sPLS-DA was run using 5-fold (community profiles and marsupial-only gene profiles) or 10-fold (comparison species gene profiles) cross-validation with 50 repeats, using the “tune” function to select the number of taxa to include. Training set samples for the prediction model are listed in Table S2. The “predict” function was used to provide methane group predictions for the test samples using the Mahalanobis distance option.

Boxplots and barcharts were created using ggplot2 v.3.5.1 (67). Heatmaps were created using pheatmap v.1.1.12 (68). Phylogenetic tree figures were created using iTOL (69).

## RESULTS AND DISCUSSION

### Marsupial species harbor diverse and distinct microbial fecal communities

**Fig 1:**
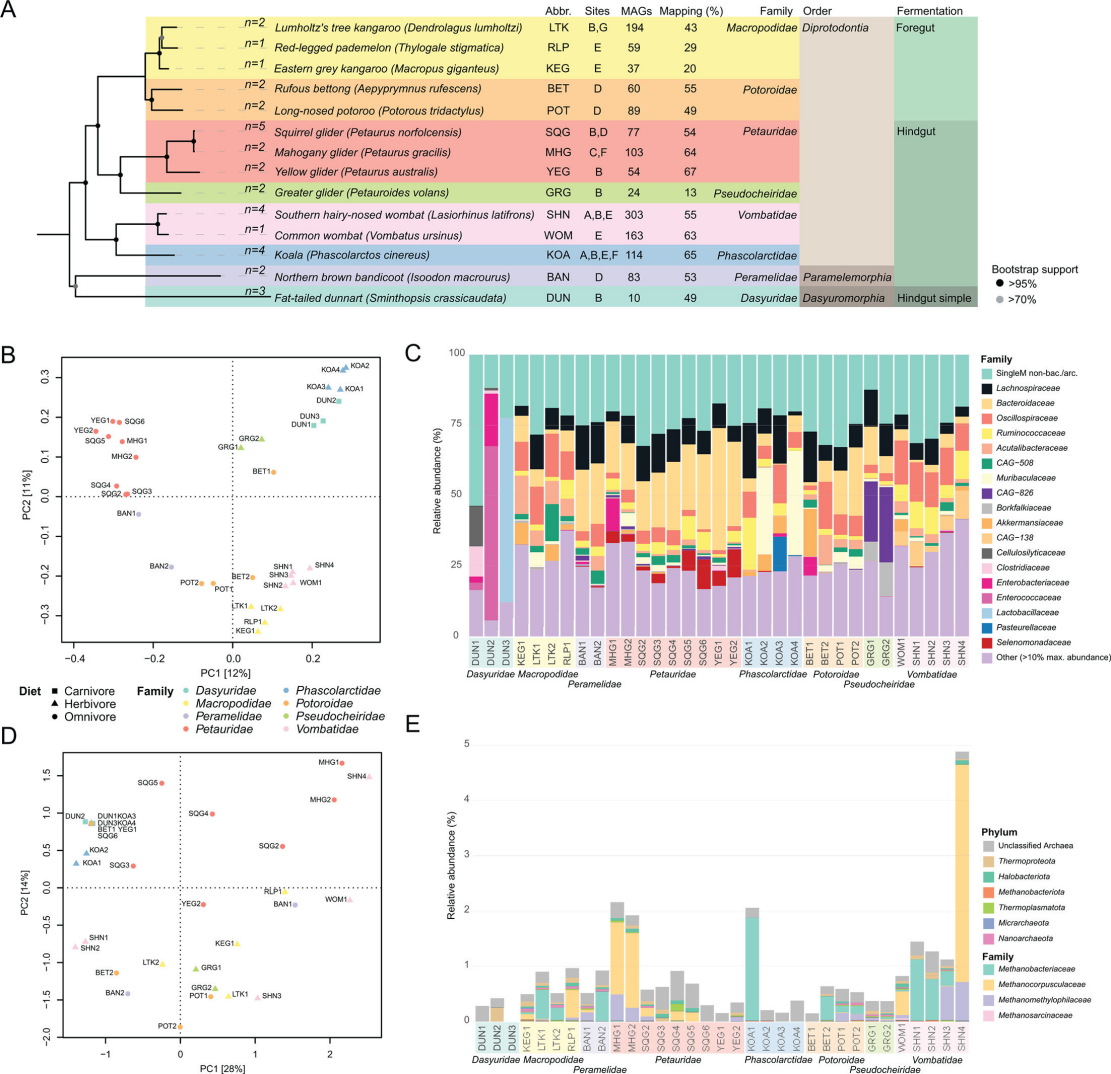
Marsupial host tree and marker gene-based community profiles. (**A**) Maximum-likelihood tree of marsupial hosts included in the data set based on alignment of two mitochondrial (*CYTB, ND2*) and three nuclear (*IRBP, BRCA1, vWF*) genes. Bootstrap support generated from 100 replicates. Sample numbers per host indicated at tips. Abbr: abbreviated host names used throughout. Site: sites sampled, (A) Cairns Tropical Zoo, (B) Currumbin Wildlife Sanctuary, (C) David Fleay Wildlife Park, (D) Hidden Vale Research Station, (E) Lone Pine Koala Sanctuary, (F) Wildlife Habitat, (G) Wongabel State Forest (wild). MAGs: number of metagenome-assembled genomes per host. Twenty-four genomes were recovered from fecal samples of two co-inhabiting species and are not included in host-level totals. Mapping (%): average percentage of sample reads mapping to the dereplicated genome database per host. (B) PCA of fecal bacterial community. (**C**) Bacterial and archaeal fecal community relative abundance at family level based on marker gene-based profiles. (**D**) PCA of fecal archaeal community. (**E**) Archaeal community family-level relative abundance based on marker gene-based profiles.

The bacterial and archaeal communities of 14 Australian marsupial species from eight marsupial families were profiled through metagenomic sequencing of 33 fecal samples (Fig. 1A and Table 1; Table S1). In addition to short-read sequencing of all samples, long-read sequencing was completed on a subset of five, selected for their identified carriage of archaeal species of interest and sequenced using adaptive sampling intended to enrich for target species. The data set produced 1,394 MAGs ≥50% complete with ≤5% contamination, 1,105 of these solely from short-read data and 289 from the hybrid assembly of short- and long-read data (Table S8). These MAGs, in addition to 1,100 public genomes estimated to represent taxa in our samples, were incorporated into a dereplicated genome database of 1,584 sequences (95% average nucleotide identity) (Fig. S1). Mapping of metagenomic sequence data to this database recruited an average of ~54% of sample reads (min. 11%, max. 82%), indicating that the sequencing and assembly effort captured a large proportion of the community, though some species were underrepresented (e.g., greater glider), resulting in poor discrimination between some hosts using principal component analysis (Table S1; Fig. S2). Up to 78% of the unrecruited sample reads (range 17%–78%, average 51%) could be aligned to contigs not included in the 1,394 MAGs obtained in the present study (Fig. S3). Bacterial species lacking genome representation comprise a substantial fraction of the unclassified data in some hosts, including the greater glider, where ~60% of unclassified reads mapped to bacterial contigs (Fig. S3). Non-prokaryote DNA was estimated to account for between 22% and 54% of total sample DNA based on marker gene assessment (Table S1), suggesting enrichment of viruses and eukaryotic microbes in some samples; however, we found limited representation of this fraction within the assembled data (Fig. S3).

Due to the incomplete representation of the fecal prokaryote community in some hosts using the combined reference genome database, the marsupial microbial communities were compared using SingleM (57). These data support a diet-associated distinction of the bacterial community, with arboreal omnivores separated from terrestrial omnivores, herbivores, and more specialized feeders (Fig. 1B), driven by the genera *Prevotella*, *Candidatus* Faecousia, and *Muribaculaceae CAG-873,* respectively (Fig. S4). The only carnivorous species in the data set, the fat-tailed dunnarts, carry distinct communities, both in comparison to other marsupials and to each other (Fig. 1C). Similarly, koala samples were also notably distinct from other marsupials and from each other (Fig. 1C). By contrast, the archaeal community varied between individual animals, and no clear separation in association with sample metadata was seen (Fig. 1D and E), as seen in marsupial fecal fungal communities (70). The bacterial dominance in driving microbial variation was also evident when considering species incidence (Fig. S5). Redundancy analysis supported the interrelated variables of host species (*R*^2^: 39%) and family (37%), gut anatomy (foregut/hindgut/simple hindgut) (9%), diet (omnivore/herbivore/carnivore) (12%), and location (5%) as contributing significantly to bacterial community-based sample divergence when each was considered in isolation (location *P* = 0.03, others *P* < 0.001). No significant variables were identified for the archaeal community profiles.

### Hydrogen management strategies in marsupials vary between and within species

**Fig 2:**
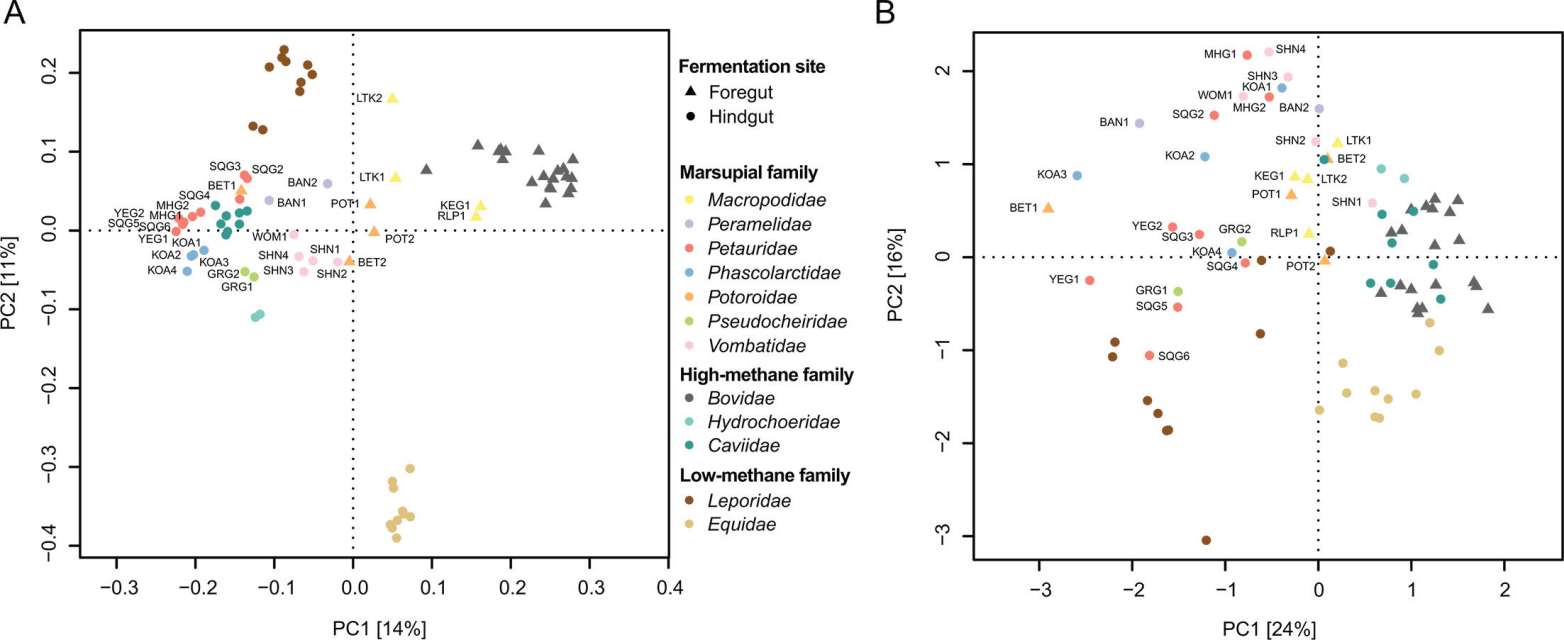
Comparison of fecal prokaryotic communities and hydrogen-cycling functions across marsupials and comparison mammals. (**A**) PCA of marker gene-based fecal prokaryotic community profiles of marsupials and high- and low-methane-producing comparison species. (**B**) PCA of hydrogen metabolism gene RPKM abundances. Public data sets include a maximum of 10 samples per host species. Data sets included are listed in Table S2.

To interpret marsupial hydrogen management capacity in the context of known methane emission phenotypes, we incorporated publicly available metagenomic data from mammals with characterized emissions and varying gut types: cattle (high-methane, foregut fermentation, *n* = 20), capybara (high, hindgut, *n* = 2), guinea pig (high, hindgut, *n* = 8), horse (low, hindgut, *n* = 29), and rabbit (low, hindgut, *n* = 30) (Table S2) (6, 27–32). While this comparison uses fecal communities from all hosts (rather than rumen for foregut fermenters), we note that rumen and fecal communities show interdependence (71), and the fecal community remains relevant to understanding the hydrogen balance of the entire system (26). As observed in marsupials, host family was the primary driver of fecal prokaryotic community composition across these hosts (*R*^2^: 55%, *P* = 0.001). Visible separation was also apparent between foregut and hindgut fermenting species (Fig. 2A), although contributing less to overall community variation (*R*^2^: 11%, *P* = 0.001). Among host families with available methane production estimates (i.e., macropodid and potoroid marsupials plus non-marsupial hosts), both gut type (marginal *R*^2^: 14%) and methane level (marginal *R*^2^: 7.5%) independently contributed to community structure (*P* = 0.001 for both).

To profile hydrogen management strategies within these fecal microbiomes, we compared the abundance of a recognized set of hydrogen-metabolizing and reductant disposal genes (3, 18) across all samples to investigate hydrogen flow and the presence of alternative hydrogen sinks. Principal component analysis of this gene set revealed high-methane-producing species (*Bovidae*, *Caviidae*, *Hydrochoeridae*) clustered together despite their phylogenetic and physiological distinctions, suggesting convergent hydrogen management strategies associated with high methanogenic capacity (Fig. 2B). By contrast, low-methane producers were more dispersed, indicating that reduced methane production may be achieved through diverse metabolic pathways. Differential abundance comparison between marsupials and high-methane mammals identified increased abundances of methanogen-specific genes (*mcrA*) and associated hydrogenases (groups 3a, 3c, and 4h [NiFe]-hydrogenases) in the high-methane group, consistent with increased methanogenic capacity (Table S9; Fig. S6A). Marsupials, in contrast, showed increased abundance of bacterial membrane-bound group 1b [NiFe] and hydB hydrogenases, which are associated with bacterial H_2_ uptake, suggesting alternative hydrogen disposal pathways to methanogenesis. We also observed a subset of marsupial samples with intermediate profiles, suggesting heterogeneity in hydrogen management strategies among marsupials (Fig. S6A). To investigate this, we used an sPLS-DA model trained on the comparison set of mammalian hosts (non-marsupials) to classify individual marsupial samples as having “high” or “low” methane-producing potential, as well as test samples from the comparison species (Fig. S6B). This analysis identified a group of marsupials classified as “high” methane producers, including all foregut fermenter samples (excepting one bettong, BET1), plus samples from hindgut-fermenting wombats (SHN1 and 3), gliders (MHG2 and SQG2), and a bandicoot (BAN2) (Table S10). Comparison of high- and low-methane marsupial groups supported enrichment of methanogenesis-associated genes within the high-methane group (Table S11; Fig. S6C), consistent with the enrichment profile observed in high-methane-producing mammals (Table S9). Of note, our data suggest possible within-species diversity of hydrogen management, as is seen in other mammals (23, 72). The low-methane group displayed a higher abundance of oxygen-tolerant (group 1d) and sensitive (groups 1b and 1i) H_2_-uptake hydrogenases that can support anaerobic respiration (73–75), as well as genes suggesting use of alternative electron acceptors within nitrate/nitrite reduction (napA, nrfA), nitrogen fixation (nifH), and sulfite reduction (asrA) (Table S10). Enrichment of these alternative hydrogen sinks in some marsupials supports reduced availability of substrates for methanogenesis in these animals (18, 76–78). These findings mirror those comparing the rumen microbiome of low- to high-methane-emitting cattle, which identified enrichment of butyrate, glutamate, and nitrate ammonification genes in low-methane-producing cattle (18).

### Marsupial methanogens are predicted to use multiple electron acceptors and carbon sources

On average, Archaea were estimated to account for <1% of the marsupial fecal prokaryotic community based on marker gene-based profiling (Table S1; Fig. 1E). The highest relative abundance (~5%) was observed in one southern hairy-nosed wombat (SHN4; Fig. 1E), dominated by *Methanocorpusculum vombati* at 3% of the total community (Table S12). Larger archaeal communities were also present in both mahogany gliders (dominant species *Methanocorpusculum petauri*) and one koala (dominant *Methanosphaera* species). Animals with high archaeal abundance typically had communities dominated by either *Methanocorpusculum* spp. or *Methanobacteriaceae* spp. (typically *Methanobrevibacter* spp.) suggestive of competition between these lineages, as previously noted (17). The most prevalent archaeal species was *Methanocorpusculum petauri*, present in 21% of samples, the majority of which originated from namesake *Petauridae* hosts (17), followed by *Methanocatella sp900769095* (15%, previously *Methanobrevibacter_A sp900769095* [79]), *Methanocorpusculum sp001940805* (12%), and a novel species belonging to the family *Methanomethylophilaceae* (*UBA71* sp., *Candidatus* Methanoprimaticola [80]). Most methanogenic archaea were identified in one or two host families, with only *Methanocatella sp900769095* found in three, suggestive of host specificity, as previously observed in other animal gut microbiomes (81). *Vombatidae* hosts carry the highest diversity of methanogens, followed by members of the families *Petauridae* and *Macropodidae*. However, we note that the diversity of identified species per host in this study differs from previous analyses. For example, we did not identify *Methanobrevibacter* or *Methanocorpusculum* species in koala, in contrast to previous studies (16, 17). The limited sample size and observed high variability between individual animals may contribute to these differences.

**Fig 3:**
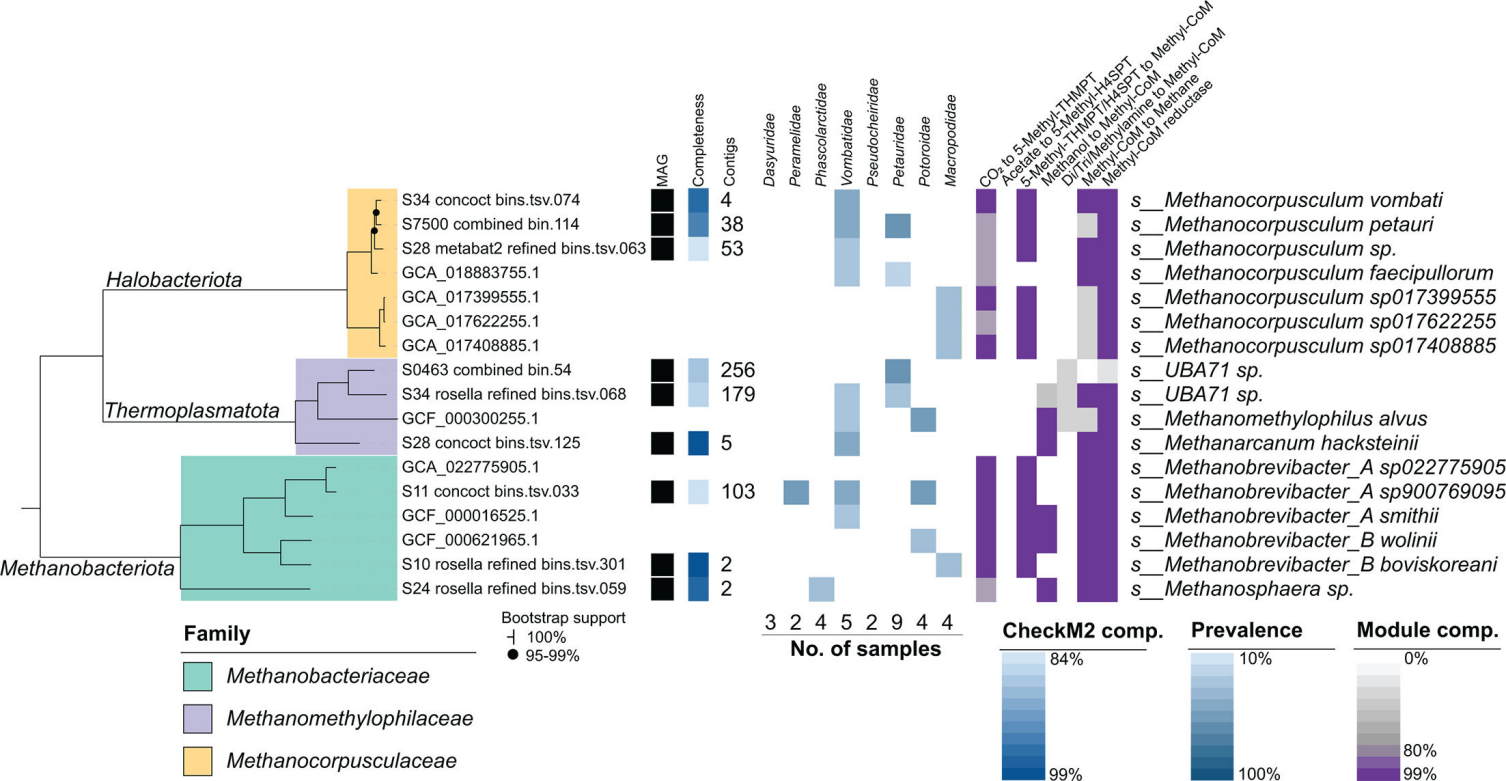
Archaeal genome tree. Maximum-likelihood phylogenetic tree inferred from alignment of 53 GTDB single-copy marker genes (43). Includes genomes from the dereplicated database, plus species-representative genomes for SingleM-identified species not represented in genome database. Presence in each marsupial host family indicated by colored boxes and determined based on sample read mapping to genome database, with presence cutoffs of >0.05% relative abundance plus >10% genome coverage. Methanogenesis pathway module completeness indicated for the representative genome of each species based on KEGG database annotation.

To gain a better understanding of the metabolic capabilities of marsupial methanogens, we reconstructed 38 MAGs supported by targeted long-read sequencing and metabolically annotated them. This revealed genus-level specialization in substrate preference as previously described (82–85) (Fig. 3; Fig. S7). The hydrogenotrophic methanogenesis pathway was largely complete in *Methanobrevibacter*, *Methanosphaera,* and *Methanocorpusculum* spp., although conservation varied between genomes, particularly for *Methanocorpusculum* representatives (Fig. S7). The methanol-based methylotrophic methanogenesis pathway was identified in *Methanosphaera* spp. and *Methanarcanum hacksteinii*, human-derived representatives of the latter previously noted for their lack of complete methanogenesis pathways (80). The *Ca*. Methanoprimaticola genus MAGs also encoded complete methylotrophic methanogenesis pathways using methanol and di/tri/methylamine, although with non-canonical methyltransferases. There was evidence for ethanol-based methanogenesis in *Methanobrevibacter_B boviskoreani* MAGs (86), with additional alcohol and aldehyde dehydrogenases identified in most genomes. Formate use was also predicted for *Methanocorpusculum* and *Methanobrevibacter* spp. Altogether, these findings suggest that small but taxonomically and metabolically diverse populations of methanogens inhabit marsupials and use both hydrogen and one-carbon substrates to drive methane production.

### Diverse hydrogenotrophic respiratory and acetogenic bacteria inhabit marsupials

Finally, we used the compiled genome database to profile the identities and functions of the putative hydrogenotrophic bacteria inhabiting marsupials. Thirty-three species encode membrane-bound H_2_-uptake hydrogenases (groups 1a–d, 1f, 1i [NiFe]-hydrogenases) that input electrons into the respiratory chain (Table S13). As expected, these species include *Desulfobacterota* (e.g., *Bilophila*, *Desulfovibrio*, group 1a) encoding the pathway for dissimilatory sulfate reduction (i.e., Apr and Dsr enzymes), with hydrogenotrophic nitrate-reducing *Campylobacter_D* also detected (group 1b). Also present were 11 species predicted to mediate hydrogenotrophic dissimilatory nitrate reduction to ammonium (DNRA, via Nar and Nrf enzymes [87]), including *Actinomycetota* species *Eggerthella lenta* and *Raoultibacter massiliensis*, and *Desulfobacterota* species *Bilophila wadsworthia* and *Desulfovibrio piger*. Other inferred hydrogenotrophs include *Parabacteroides*, *Megamonas*, *Parasutterella*, *Akkermansia*, and the uncultivated *Planctomycetota* genus *RUG369*. Culture-based experiments are needed to confirm the role of hydrogen uptake in each of these genera.

While acetate production has been confirmed as a primary output of macropod foregut communities (88, 89) and is considered the dominant H_2_ disposal mechanism in these animals (90), little is known about potential acetogenic populations across other marsupials. This trait appears to be widespread, with 70 species from 23 genera encoding both the *acsB* and *cooS* genes, the key determinants for acetogenesis, the acetyl-CoA synthase/carbon monoxide dehydrogenase complex (Table S13). Most marsupial hosts harbored multiple putative acetogenic genera, except for the greater glider, which was underrepresented in the genome database, and the macropods, carrying solely *Ca*. Faecousia species. Proposed association of acetogens with epithelial-attached biofilms in the macropodid forestomach (90) may limit their recovery via fecal sampling. All predicted acetogens affiliated with five families of *Clostridia*, except for the putative flagellate-associated acetogen *Candidatus* Adiutrix from the phylum *Desulfobacterota* (91). MAGs were obtained for the well-characterized acetogenic genus *Blautia* as well as numerous uncultivated lineages, most notably multiple MAGs of *Ca*. Faecousia and *CAG-170* that was previously shown to become enriched in ruminants following inhibition of methanogenesis, indicating a shift toward H_2_ disposal via acetogenesis with this treatment (25). Almost all (90%) of the 70 species encode [FeFe]-hydrogenases from group A (29 species) and/or B (57 species), suggesting that they can use H_2_ to reduce CO_2_ to acetate. It is unclear how acetogens co-exist and potentially outcompete methanogens in marsupials given their higher H_2_ threshold and lower energy yield (92). One possibility is that these acetogens adopt a mixotrophic metabolism, using a combination of organic and inorganic substrates, including direct formate assimilation rather than using the hydrogen-dependent CO_2_ reductase reaction, as recently described (25). It is also possible that the spatial organization of marsupial guts provides a range of niches that enable co-existence of acetogens and methanogens.

### Conclusion

This study provides the first functional characterization of hydrogen management across marsupial gut microbiomes, revealing diversity in predicted methanogenic capacity across both species and gut types. Our analysis indicates that marsupials span a functional spectrum: some harbor methanogen-rich communities with elevated methanogenesis genes, while others show enrichment of competing bacterial processes, including hydrogenotrophic respiration and reductive metabolism. This functional heterogeneity provides a mechanistic basis for understanding variation in methane production potential across marsupial lineages, providing hypotheses to be validated through activity-based measurements.

## Data Availability

The data sets generated during the current study are available in the NCBI repository under BioProject PRJNA1167890. Additional sequencing data were produced for three previously published samples: S29, S30, and S34 (17) (Table S1). MAGs over 90% complete are available via NCBI with the complete set available at figshare 10.6084/m9.figshare.27168006.v1.

## SUPPLEMENTAL MATERIAL

Supplemental Figures msystems.01608-25-s0001.pdf Figures S1 to S7.

Supplemental Tables msystems.01608-25-s0002.xlsx Tables S1 to S12.

ASM does not own the copyrights to Supplemental Material that may be linked to, or accessed through, an article. The authors have granted ASM a non-exclusive, world-wide license to publish the Supplemental Material files. Please contact the corresponding author directly for reuse.
