# Supplementary material for: Metagenomic analysis of fecal microbiomes reveals genetic potential for diverse hydrogen management strategies in marsupials
Source: mSystems. 2025 Dec 23;11(2):e01608-25. doi: 10.1128/msystems.01608-25 (PMC12911397; doi:10.1128/msystems.01608-25)
Supplement: Supplemental Figures — Figures S1 to S7. [file msystems.01608-25-s0001.pdf]

Supplementary Figures

Metagenomic analysis of faecal microbiomes reveals genetic potential for diverse hydrogen management strategies in marsupials

Kate L. Bowerman[1], Yang Lu[2], Harley McRae[3], James G. Volmer[4], Julian Zaugg[1], Phillip B. Pope[4 5], Philip Hugenholtz[1], Chris Greening[6], Mark Morrison[3], Rochelle M. Soo[1], Paul N. Evans[1]*

[1] Australian Centre for Ecogenomics, School of Chemistry and Molecular Biosciences, The University of Queensland, Brisbane, Australia
[2] Water Innovation and Smart Environment Laboratory, School of Civil and Environmental Engineering, Faculty of Engineering, Queensland University of Technology, Brisbane, Australia
[3] Frazer Institute, Faculty of Medicine, The University of Queensland, Brisbane, Australia
[4] Centre for Microbiome Research, School of Biomedical Sciences, Queensland University of Technology, Brisbane, Australia
[5] Faculty of Chemistry, Biotechnology and Food Science, Norwegian University of Life Sciences, Ås, Norway
[6] Department of Microbiology, Biomedicine Discovery Institute, Monash University, Clayton, Australia

* Corresponding author

Figure S1

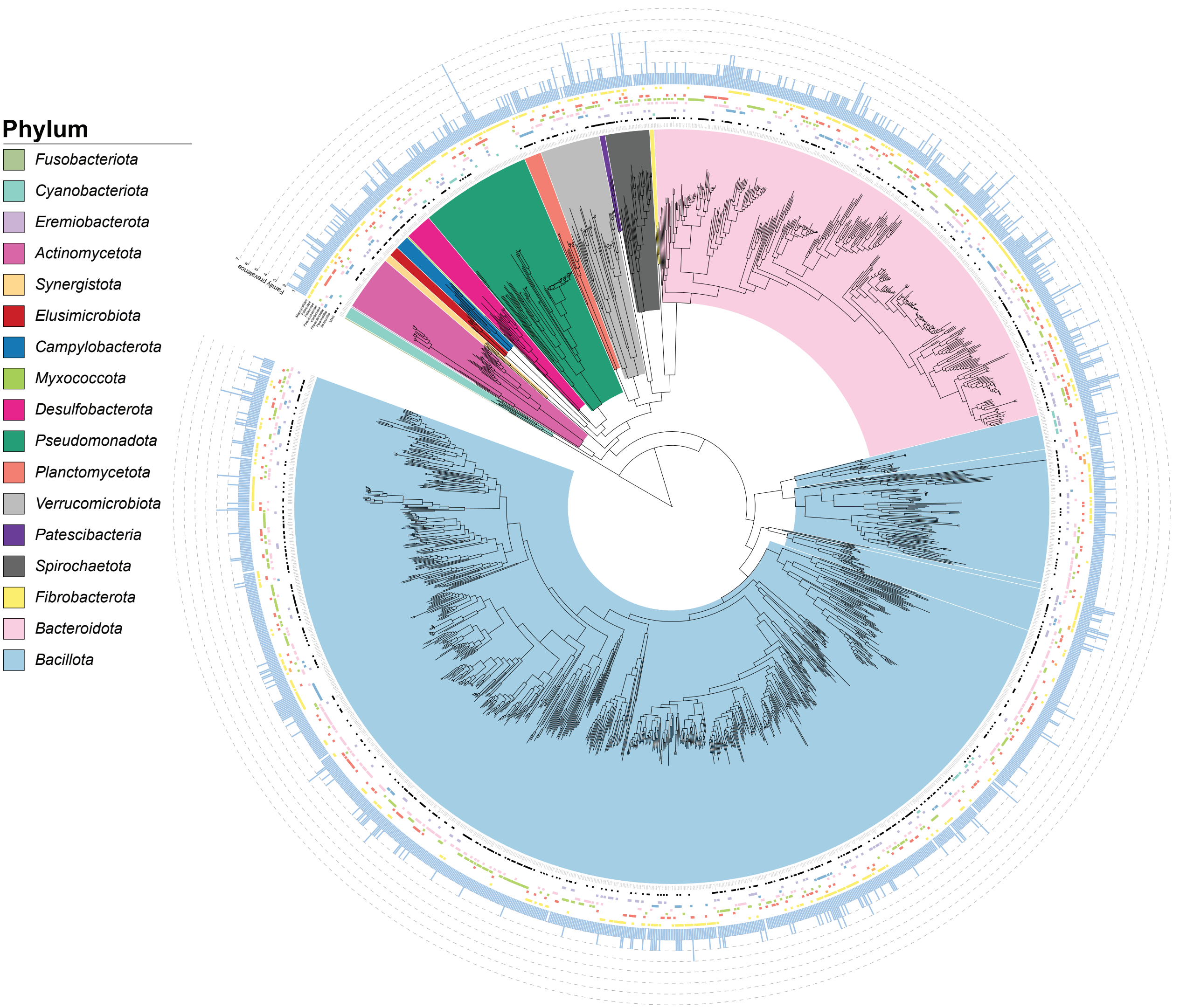

Fig. S1. Bacterial genome tree. Maximum likelihood phylogenetic tree inferred from alignment of 120 GTDB single-copy marker genes [40,41]. Includes genomes from the dereplicated database plus species representative genomes for SingleM-identified species not represented in genome database. Outer rings represent presence in each marsupial host family determined based on sample read mapping to genome database with presence cutoffs of >0.05% relative abundance plus >10% genome coverage. Outermost ring displays count of host families where at least one sample met this threshold.

Figure S2

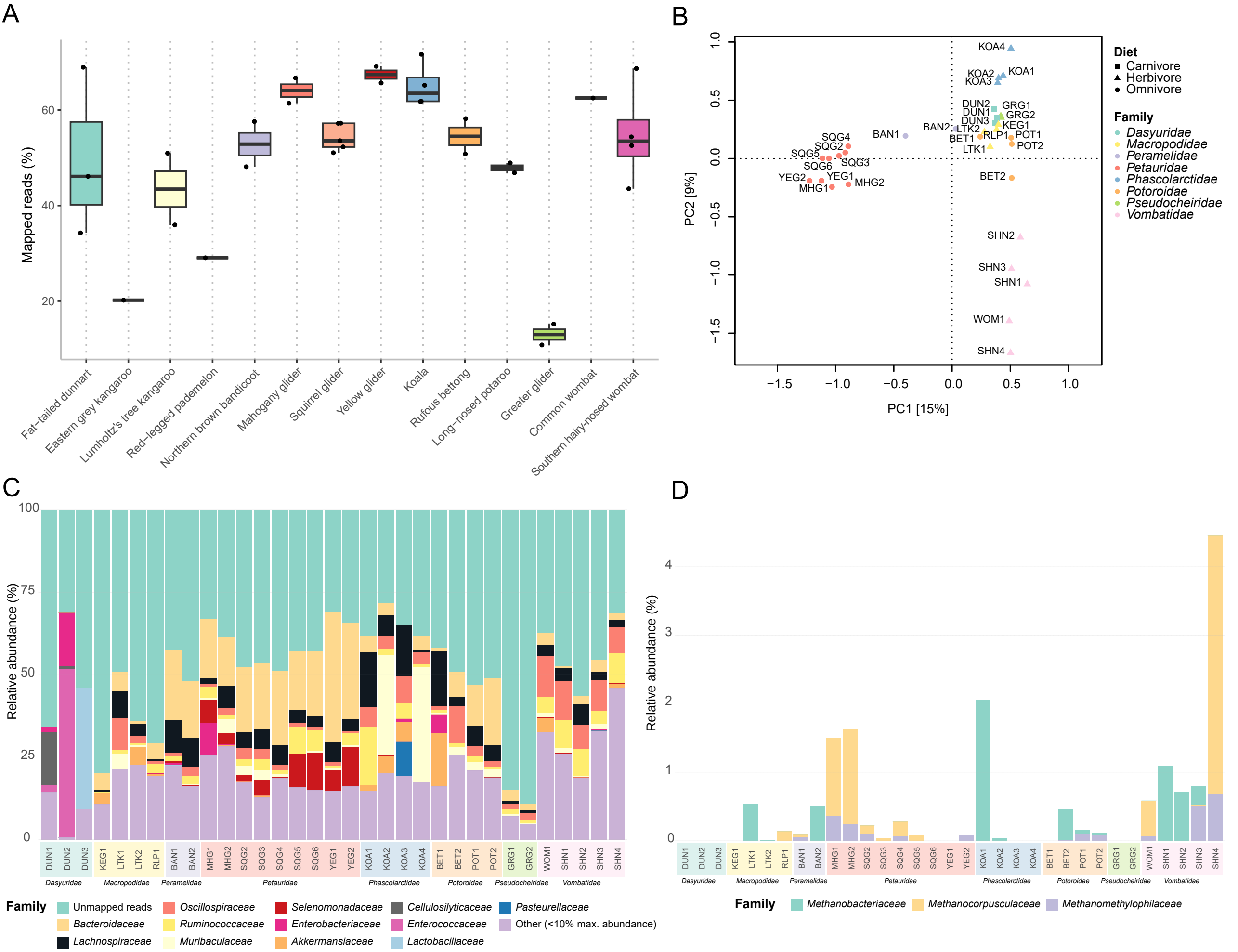

Fig. S2. Genome-based community composition. (A) Read mapping to the dereplicated genome database, filtering for alignments >95% identical across >90% of the read length. (B) Principal component analysis based on square-root transformed relative abundance values. Family level relative abundance of bacterial (C) and archaeal communities (D). Host species are abbreviated as DUN: fat-tailed dunnart, KEG: eastern grey kangaroo, LTK: Lumholtz’s tree kangaroo, RLP: red-legged pademelon, BAN: northern brown bandicoot, MHG: mahogany glider, SQG: squirrel glider, YEG: yellow glider, KOA: koala, BET: rufous bettong, GRG: greater glider, WOM: common wombat, SHN: southern hairy-nosed wombat.

Figure S3

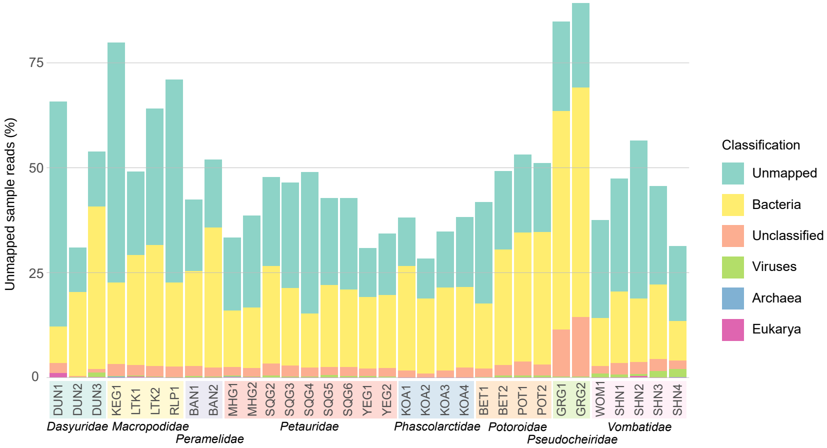

Fig. S3. Classification of unmapped sample reads. Sample reads not aligning to the genome database were mapped to assembled contigs not included within study MAGs. Contigs were classified to domain level using MMseqs2 [49] to estimate fractional representation of each domain within the unmapped data. Classification 'Unmapped' denotes reads that did not align to the unbinned contigs. Host species are abbreviated as DUN: fat-tailed dunnart, KEG: eastern grey kangaroo, LTK: Lumholtz's tree kangaroo, RLP: red-legged pademelon, BAN: northern brown bandicoot, MHG: mahogany glider, SQG: squirrel glider, YEG: yellow glider, KOA: koala, BET: rufous bettong, GRG: greater glider, WOM: common wombat, SHN: southern hairy-nosed wombat.

Figure S4

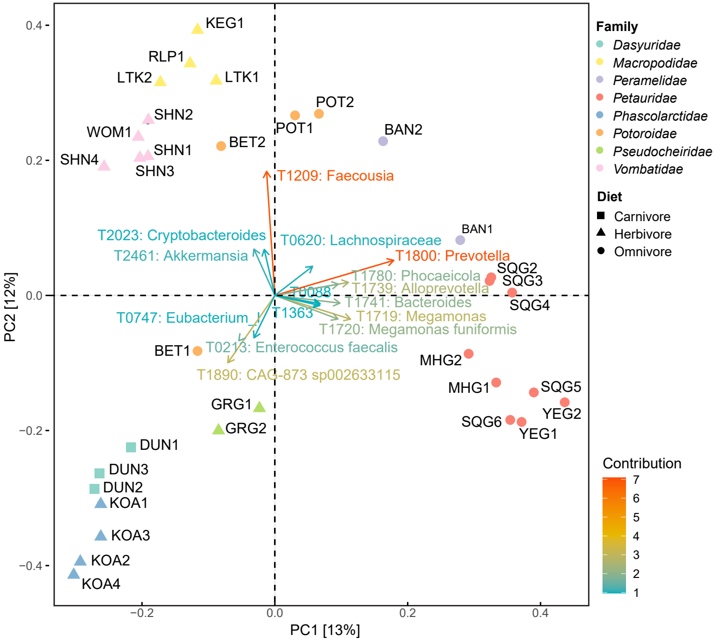

Fig. S4. Taxa contributing to marker gene-based community profile components. Principal components analysis of faecal bacterial and archaeal community displaying the top 15 taxa contributing to sample separation. Host species are abbreviated as DUN: fat-tailed dunnart, KEG: eastern grey kangaroo, LTK: Lumholtz's tree kangaroo, RLP: red-legged pademelon, BAN: northern brown bandicoot, MHG: mahogany glider, SQG: squirrel glider, YEG: yellow glider, KOA: koala, BET: rufous bettong, GRG: greater glider, WOM: common wombat, SHN: southern hairy-nosed wombat.

Figure S5

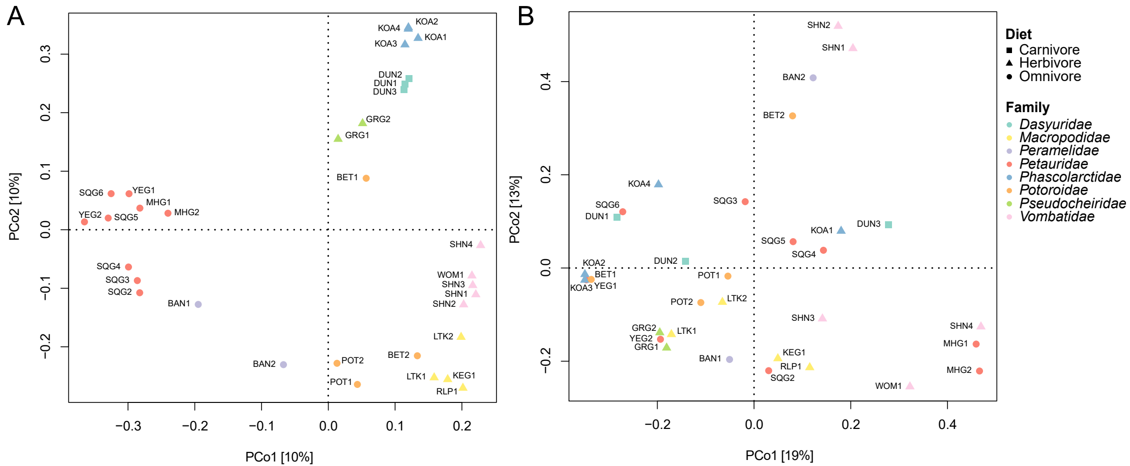

Fig. S5. Marker gene-based community presence/absence profiles. Principal coordinates analysis of faecal bacterial (A) and archaeal (B) community presence/absence (Jaccard distance). Host species are abbreviated as DUN: fat-tailed dunnart, KEG: eastern grey kangaroo, LTK: Lumholtz's tree kangaroo, RLP: red-legged pademelon, BAN: northern brown bandicoot, MHG: mahogany glider, SQG: squirrel glider, YEG: yellow glider, KOA: koala, BET: rufous bettong, GRG: greater glider, WOM: common wombat, SHN: southern hairy-nosed wombat.

Figure S6

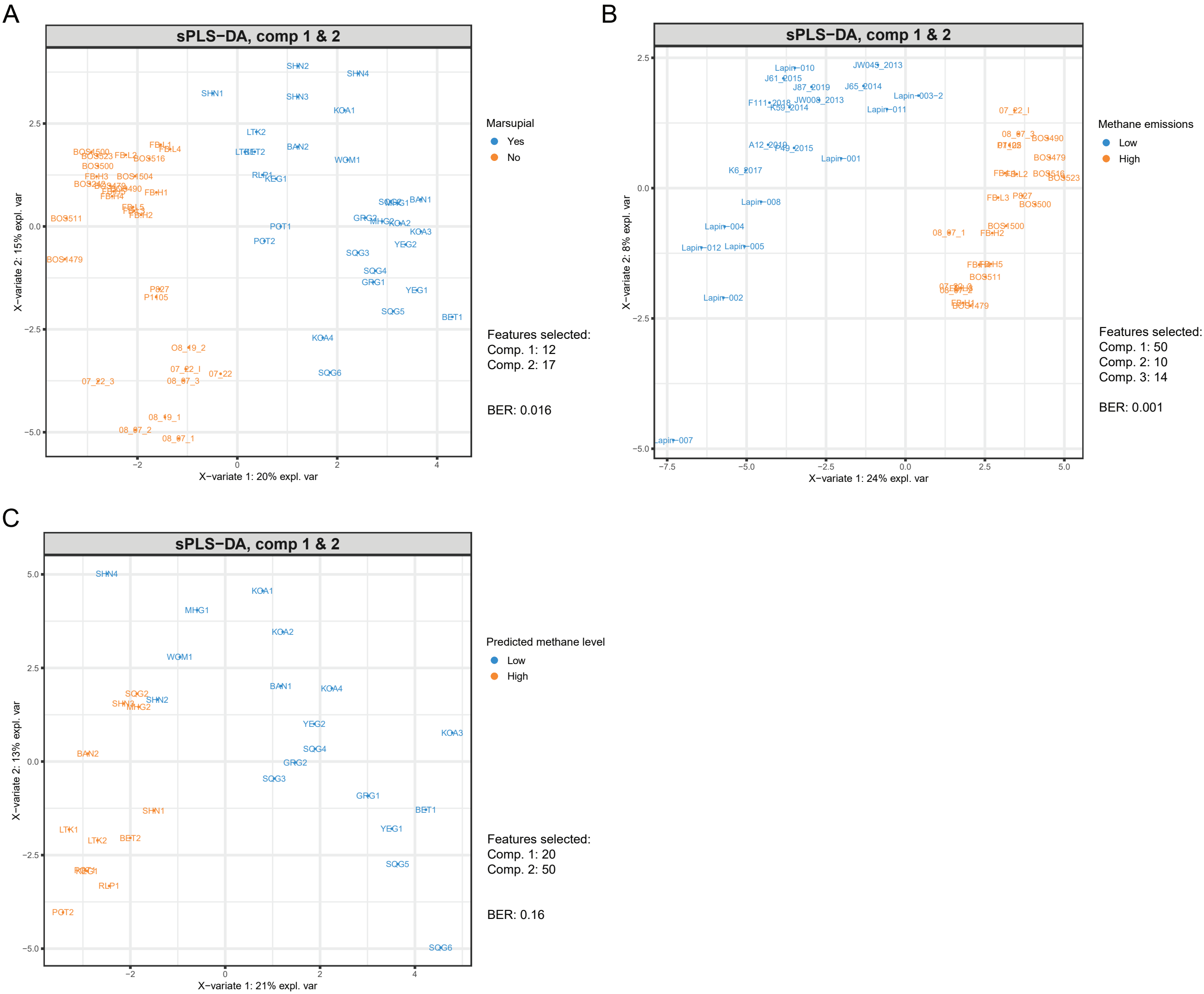

Fig. S6. sPLS-DA based on hydrogen-cycling gene abundance across faecal samples. Models include A) non-carnivorous marsupials and high-methane emitting comparative species, classification variable: marsupial vs non-marsupial, B) training set of high- and low-methane emitting comparative species, classification variable: methane emission level, and C) non-carnivorous marsupials, classification variable: predicted methane status from model in B.

Figure S7

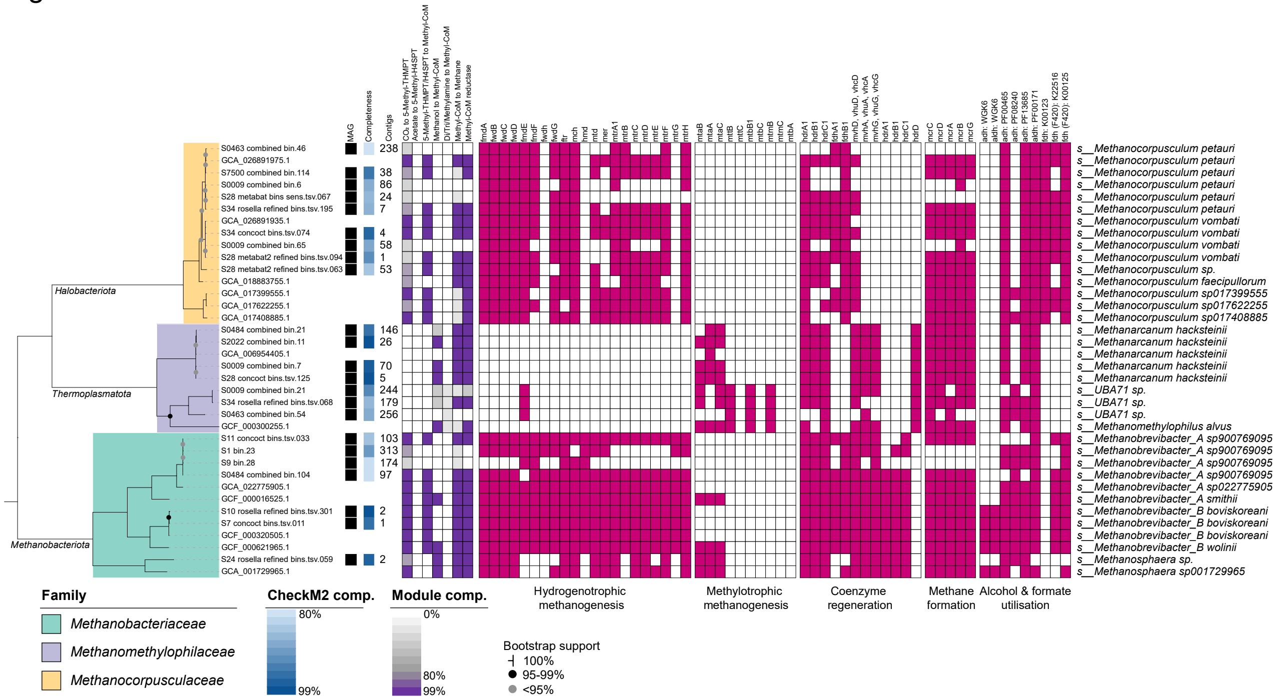

Fig. S7. Methanogenesis gene presence across archaeal genomes. Maximum likelihood phylogenetic tree inferred from alignment of 53 GTDB single-copy marker genes. Includes genomes assembled from the dataset exceeding 80% completeness, dereplicated at 80% identity. Supplemented with public genomes representing species present in the dataset or derived from other marsupial studies [15, 83]. Methanogenesis pathway module completeness and individual gene presence indicated for each genome based on KEGG database annotation. Alcohol and formate dehydrogenase presence derived from BLAST-determined homology to WGK6 proteins [83], Pfam or KEGG annotations as indicated.
